# Referral determinants in Swiss primary care with a special focus on managed care

**DOI:** 10.1371/journal.pone.0186307

**Published:** 2017-11-07

**Authors:** Ryan Tandjung, Seraina Morell, Andreas Hanhart, Andreas Haefeli, Fabio Valeri, Thomas Rosemann, Oliver Senn

**Affiliations:** 1 Institute of Primary Care, University and University Hospital of Zurich, Zurich, Switzerland; 2 Private Primary Care Practice, Wetzikon, Switzerland; 3 Private Primary Care Practice, Lupfig, Switzerland; National Yang-Ming University, TAIWAN

## Abstract

Studies have shown large variation of referral probabilities in different countries, and many influencing factors have been described. This variation is most likely explained by different healthcare systems, particularly to which extent primary care physicians (PCPs) act as gatekeepers. In Switzerland no mandatory gatekeeping system exists, however insurance companies offer voluntary managed care plans with reduced insurance premiums. We aimed at investigating the role of managed care plans as a potential referral determinant in a non-gatekeeping healthcare system. We conducted a cross-sectional study with 90 PCPs collecting data on consultations and referrals in 2012/2013. During each consultation up to six reasons for encounters (RFE) were documented. For each RFE PCPs indicated whether a referral was initiated. Determinants for referrals were analyzed by hierarchical logistic regression, taking the potential cluster effect of the PCP into account. To further investigate the independent association of the managed care plan with the referral probability, a hierarchical multivariate logistic regression model was applied, taking into account all available data potentially affecting the referring decision. PCPs collected data on 24’774 patients with 42’890 RFE, of which 2427 led to a referral. 37.5% of patients were insured in managed health care plans. Univariate analysis showed significant higher referral rates of patients with managed care plans (10.7% vs. 8.5%). The difference in referral probability remained significant after controlling for other confounders in the hierarchical multivariate regression model (OR 1.355). Patients in managed care plans were more likely to be referred than patients without such a model. These data contradict the argument that patients in managed care plans have limited healthcare access, but underline the central role of PCPs as coordinator of care.

## Introduction

A strong primary care contributes to quality and efficiency of a healthcare system [1]. This crucial role of primary care physicians (PCP) is reflected in referrals from primary to secondary care. Studies have shown large variation of referral probabilities in different countries besides many other influencing factors such as age and sex of patients as well as sex of physicians, [2–5].

The variation between countries might be most likely explained by different healthcare systems; the extent to which PCPs act as gatekeepers and the revenue scheme for the health care providers are important determinants. In healthcare systems with a strong gatekeeper role of PCPs (Norway and in the United Kingdom for example) referral rates are higher than in the United States [4, 6, 7]. The role of managed health care plans has so far only rarely been assessed, mainly due to the fact that in many healthcare settings the freedom of choice between different systems is not given. Forrest [8] showed that patients in managed health care plans in the US were more likely to be referred than patients in non-gatekeeping plans. In Switzerland, health insurance is compulsory for all citizens and is financed by health insurance premiums. In the Swiss healthcare system, no mandatory gatekeeping mechanisms exists. However, the insured person can voluntarily choose a managed health care plan implying a gatekeeping system, with the benefit of a premium reduction. In 2013 21% of the Swiss population were insured in managed health care plans [9]. The introduction of mandatory managed health care plans for all citizens was highly rejected by a public vote in 2012; a major fear was that gatekeeping results in restrictions to healthcare access and in reduced health care quality.

In Switzerland, systematic data on referrals was last collected in 1989 within a European referral study [10]. Many circumstances have changed since then, including compulsory health care insurance for every Swiss citizen since 1996 and the introduction of managed health care plans. So far, the impact of these changes on referral rates is unclear. We aimed at investigating which factors influence referral rates from primary to secondary care, with a special focus on voluntary managed care plans as a potential referral determinant in a non-gatekeeping healthcare system.

## Materials and methods

This study is based on the Swiss referral study with previously reported details regarding methods and referral rates [11]. In summary, we prospectively collected data of consultations during maximally 15 days of 92 PCPs. Every consultation was recorded with a small set of patient data (age and sex, membership in a managed care health plan), and up to six reasons for encounter (RFE) for each consultation, since patients often have more than one RFE per consultation [12]. For each RFE, PCPs indicated whether a referral was initiated. We collected age and sex of PCPs, years of experience as PCP, working condition such as number of working days per week, and practice form (single-handed, double practice or group practice). Data was collected during three different months (November, March and Mai) in 2012/2013. During each month, every weekday was represented once.

Furthermore, PCPs completed two validated questionnaires concerning the handling of uncertainty in primary care (dealing with uncertainty questionnaire and physicians reaction to uncertainty scales) [13, 14]. The questionnaire on dealing with uncertainty has two categories (action scale and diagnostic reasoning scale ranging from 1 to 6, with 6 indicating “higher concerns”). The questionnaire on physicians’ reaction to uncertainty scales has four categories ranging from 1 to 6, with 6 indicating “higher uncertainty”: Anxiety due to uncertainty, concerns about bad outcomes, reluctance to disclose to patients and reluctance to disclose to physicians.

### Statistics

Demographic data of patients and PCPs was analyzed with descriptive statistics and is presented in means and standard deviation or percentages. Univariate comparisons between groups were analyzed by means of chi-square or t-tests. The sum scores of the subcategories were included into our multivariable regression model. Statistical analysis was performed with R, version 3.1.2 [15].

In the multivariable regression model, referral probability was the independent variable, dependent variables were: patient determinants (age, sex, managed care model), PCP determinants (sex, practice form, experience in years, patient load [patients per day], workload [number of half days per week]), number of RFE, scores in uncertainty questionnaires, season, and weekday. We performed a hierarchical univariate logistic regression with PCP as random effect. To further investigate the independent association of managed care plan on referral probability, we applied a hierarchical multivariate logistic regression model, taking into account all available data potentially affecting the decision to refer and PCP as random effect. Results of the regression analysis are presented as crude and adjusted odds ratios (OR) with 95% confidence intervals (95%CI). As significance level a two-sided p value <0.05 was defined. The cluster effect was estimated by the intra-class correlation coefficient (ICC). We checked for modifying effects on the referral rate between sex of physicians and workload, average number of patients per day and experience in years, by including interaction terms in the multivariable regression model. The interactions were not significant and therefore we did not include these interactions in our final model.

### Ethical approval

According to Swiss ethics guidelines a study based on anonymous data does not require a formal approval of an Ethic committee. For the present study, we consulted the Ethics Committee of the Canton of Zurich, which confirmed that an ethical approval was not necessary (no objection of the Ethics Committee, correspondence letter from June 28th 2012). Data was treated confidentially.

## Results

### Participants

Detailed demographic data on participating PCPs is reported elsewhere [11]. Overall data of 90 PCPs was included in the study. 24,774 consultations and 42,890 RFE were recorded. 2,427 RFE (of 2,341 consultations) led to a referral, corresponding to a referral rate of 9.4%. Table 1 shows patient characteristics with respect to managed care health plans.

**Table 1:** Data on patients with and without managed care plans.

	Managed care	No managed care	P-value
Number of patients (%)	9’278 (37.5%)	15’496 (62.5%)	
Number of female patients (%)	5’196 (56.0%)	8’236 (53.1%)	p<0.001
Age in years (SD)	54.90 (21.23)	53.26 (22.09)	p<0.001
Mean number of RFE (SD)	1.82 (1.29)	1.68 (1.02)	p<0.001
Referral rate (%)	10.7%	8.5%	p<0.001

Demographic data on patients included in the study. Figures indicate absolute and relative frequencies for the number of patients and means (including standard deviation in brackets) for patients’ age and number of reasons for encounters (RFE).

Details of all determinants of the univariate and multivariate analysis are shown in Table 2. The univariate analysis showed: A positive association with managed care plan; on the patient level a significant non-linear association between patients’ age and referral probability, as well as male sex. Furthermore, the referral probability was higher in patients presenting with more RFE per consultation. On the level of PCPs, female PCPs were more likely to refer; PCPs working in double- or group-practices were associated with higher referral rates. Workload and the number of patients per day were negatively associated with the referral rate. The likelihood of referral was higher on Mondays and Fridays, compared to the other working days. No influence of season could be observed. In the uncertainty survey, three dimensions (anxiety, bad outcomes and action scale) were significantly associated with the likelihood of referral.

**Table 2:** Factors influencing referral rate.

	Univariate hierarchical analysis			Multivariate hierarchical analysis		
	OR	95%-CI	p-value	OR	95%-CI	p-value
**Patient characteristics**						
Age	1.059	(1.049–1.070)	<0.001	1.056	(1.044–1.069)	<0.001
Age2	0.999	(0.999–1.000)	<0.001	0.999	(0.999–1.000)	<0.001
Male	1			1		
Memale	0.904	(0.829–0.986)	0.023	0.884	(0.804–0.973)	0.012
Managed care (no)	1			1		
Managed care (yes)	1.355	(1.235–1.487)	<0.001	1.348	(1.221–1.488)	<0.001
**Reason for encounter**^a^						
Number of RFE	1.235	(1.183–1.289)	<0.001	1.248	(1.191–1.308)	<0.001
**PCP characteristics**						
Male	1			1		
Female	1.327	(1.079–1.632)	0.007	1.412	(1.096–1.820)	0.008
**Practice form**						
Single-handed practice	1			1		
Double practice	1.535	(1.274–1.851)	<0.001	1.587	(1.300–1.938)	<0.001
Group practice	1.273	(1.062–1.526)	0.009	1.178	(0.940–1.476)	0.155
Workload^b^	0.939	(0.897–0.983)	0.007	1.040	(0.979–1.104)	0.206
Experience^c^	0.997	(0.988–1.006)	0.456	1.003	(0.994–1.012)	0.476
Patient load^d^	0.986	(0.979–0.992)	<0.001	0.984	(0.978–0.991)	<0.001
**Other Factors**						
**Weekday**						
Monday	1.292	(1.137–1.467)	<0.001	1.321	(1.149–1.519)	<0.001
Tuesday	1			1		
Wednesday	1.087	(0.947–1.248)	0.237	1.094	(0.940–1.274)	0.245
Thursday	0.995	(0.849–1.166)	0.950	0.953	(0.799–1.138)	0.596
Friday	1.158	(1.015–1.322)	0.030	1.156	(1.000–1.336)	0.050
**Season**						
Spring	1			1		
Autumn	1.069	(0.963–1.187)	0.209	1.027	(0.916–1.152)	0.646
Winter	0.933	(0.840–1.038)	0.202	0.909	(0.810–1.021)	0.107
**Survey**^e^						
Anxiety	1.116	(1.021–1.219)	0.015	1.011	(0.916–1.115)	0.835
Bad outcomes	1.109	(1.014–1.214)	0.024	1.061	(0.957–1.176)	0.263
Disclose to patients	0.998	(0.910–1.094)	0.962	0.978	(0.904–1.057)	0.570
Disclose to physicians	1.006	(0.919–1.102)	0.897	1.042	(0.954–1.138)	0.359
Action scale	1.099	(1.006–1.200)	0.036	1.125	(1.027–1.233)	0.011
Diagnostic reasoning scale	0.961	(0.878–1.051)	0.384	0.941	(0.867–1.021)	0.142

Influencing patient and PCP characteristics on the likelihood for a referral. Figures are indicated in odds ratios (OR) with 95%-confidence intervals (95%CI) and p-values. The left columns indicate univariate regression analyses. The right columns show the results of the hierarchical multivariate regression model, controlled for all determinants presented in the table. The hierarchical analysis took into account individual patient data on the level of the PCP (cluster). The cluster effect of the multivariate regression model was ICC = 0.019. Interactions between sex of physicians and workload (p = 0.277), sex of physicians and patient load (p = 0.233) as well as sex of physicians and experience (p = 0.386) were not included in the model, because these interactions were not statistically significant.

^a^RFE, number of reasons for encounter per consultation;

^b^Workload (PCP), indicates number of half-days per week;

^c^Experience, indicates experience of PCP in years working as physicians in primary care;

^d^Patient load, number of patients / day:

^e^Survey, results are based on two questionnaires: diagnostic uncertainty questionnaire and physicians’ reaction to uncertainty

In the multivariate regression model, two factors became statistically non-significant: Anxiety and bad outcomes of the uncertainty questionnaire and, on PCP level, the setting of a group-practice. All other factors remained significant. Based on the number of consultations, the crude referral rates between PCPs varied between 1.48 to 24.2%. This variation between PCPs decreased in the multivariate regression analysis; resulting in an adjusted referral rate ranging from 5.6% to 17.4%. The ICC of the hierarchical multivariate regression model was 0.019. We tested for interactions between sex of PCPs and workload (p = 0.277), sex of PCPs and number of patients (p = 0.233) as well as sex of PCPs and experience (p = 0.386), in the final model we did not include these interactions, because they were not statistically significant.

**Fig 1:**
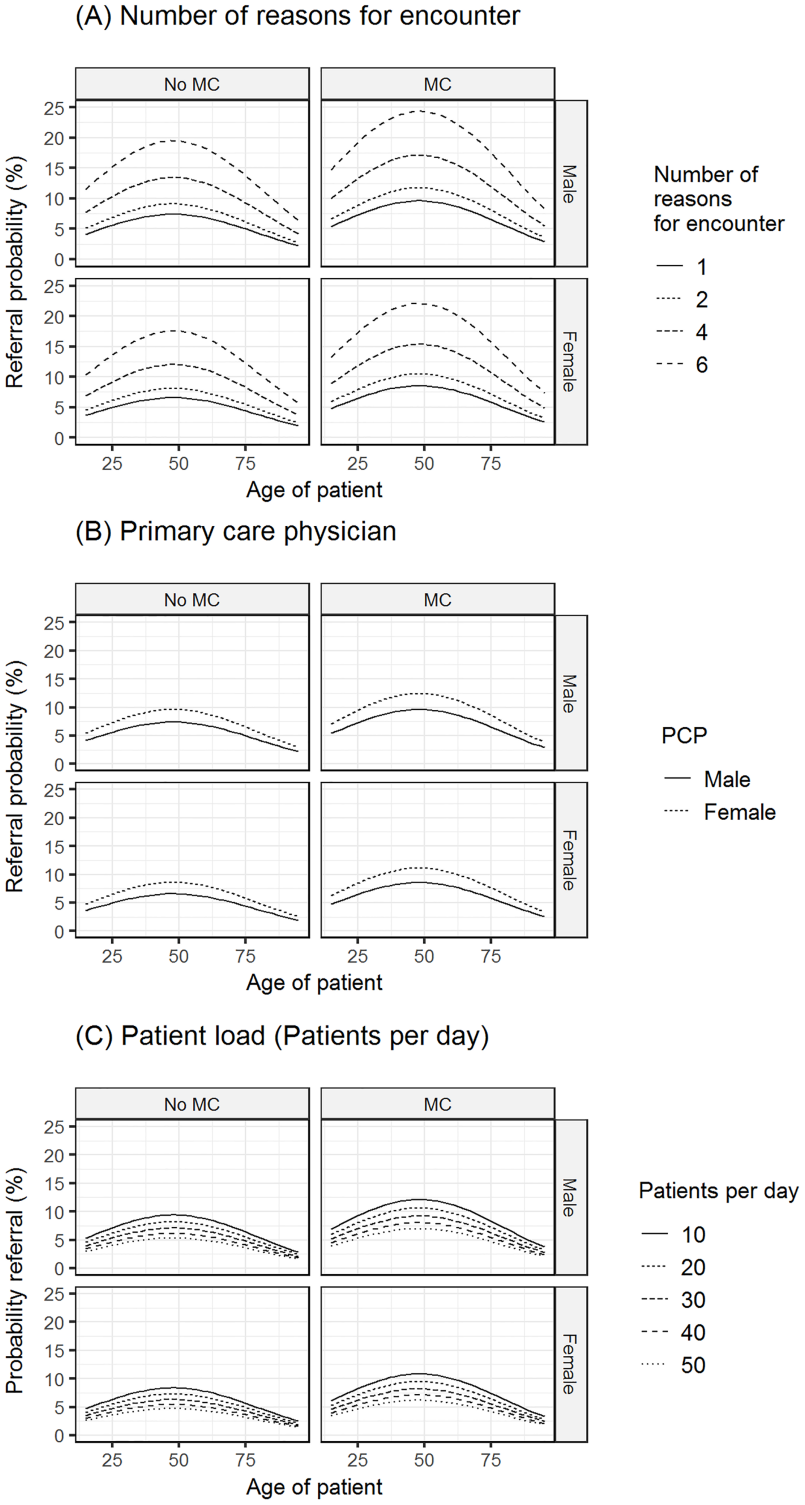
Estimated referral probabilities stratified according to the managed care status (MC). **Fig** 1 shows the estimated referral probabilities (y-axis) in relation to the patients’ age (x-axis) based on the multivariate hierarchical regression model and stratified according to the managed care status (MC). The regression model controlled for all determinants depicted in Table 2. A significant non-linear association exists between the referral probability and patients’ age, which is independent of the MC status. Panels (A) to (C) show the independent influence of different patient and PCP determinants on the referral probability (A), number of reasons for encounters, (B) sex of PCP, (C) patient load per day.

Fig 1 shows the estimated referral probabilities, based on the hierarchical multilevel model stratified according to the membership in a managed health care plan. On average being insured in a managed care plan was associated with an increased referral rate of 36% (adjusted OR 1.355; 95%-CI: 1.235–1.487).

## Discussion

We investigated the role of a managed health care plan as a referral determinant based on a large prospectively collected sample of consultations and referrals in Swiss primary care. Patients enrolled in a managed health care plan showed a referral rate that was 36% higher compared to patients without a managed health care plan. In a non-gatekeeping health care system, a voluntary managed health care plan remained a significant determinant, after correcting for several known factors influencing the referral rate.

The inclusion of several parameters in the multivariable regression model confirmed diverse known determinants: age and sex of patients [4, 16–18], providers characteristics, such as sex of PCP [3, 16, 18–20], workload [16, 18, 19, 21, 22] or practice structure [2, 3, 23, 24] and other factors such as weekday [25]. The representativeness of our data and robustness of our model was confirmed by the fact that season had no impact on the referral probability and referral rate increased with the number of RFE per consultation. One factor was different from previous findings; in our study PCPs with higher patient load had lower referral probability than PCPs with less patient encounters. Earlier studies either described higher referral probabilities with higher patient load [21, 22, 26] or no influence [16, 18]. A possible explanation might be assumed in different working patterns: some PCPs might prefer shorter but more consultations. In our model, we included the daily patient load; we were unable to include the whole patient registry, which might explain this particular difference. We found a wide range of referral rates across PCPs, which has been reported in a literature review by O’Donnell [5]. This variation is also reflected by the small cluster effect, detected in our regression analysis, indicating an unexplained influence of the PCP on the variation of the referral probability. The ICC of 1.9% shows a small influence of the provider on the variation of the referral probability, indicating that the referral probability does not just reflect a personal “referring-pattern”. The fact that of all included uncertainty scores, only the action scale of the dealing with uncertainty questionnaire [13] remained significant, also indicates that personal preferences of PCPs only had a small influence.

A specific feature in Swiss healthcare is the compulsory health insurance for every citizen. Additionally a managed health care plan with lower premiums can be voluntarily obtained. In a public vote in 2012 a majority of 76.0% voted against the implementation of mandatory managed health care plans. The main reason for the rejection was the fear of containment of services. Even though our data is limited by the fact that the amount of patients seeking a specialist directly could not be assessed, our study does not support this hypothesis. Patients with managed health care plans were more likely to be referred, which confirms earlier US data [8]. Patients with normal insurance plans are nevertheless able to seek specialist care directly without consulting the PCP. However, clinical experience and analysis of our data showing similar distribution of RFE and similar demographic data in the two groups, do not indicate that this is often the case. Furthermore, several studies have indicated that patients showed higher satisfaction when referrals were initiated by their PCP, compared to self-initiated referrals [27–29]. Even though patients in managed health care plans were more likely to be referred, Swiss and international data suggest overall lower costs of managed health care [30, 31]. In the context of cost data and similar patient characteristics in our study sample, the higher referral probability of the managed health care plan does not seem to reflect an inappropriate use of specialist care. These data indicate the central role of PCPs as coordinator of care, which results in lower overall costs and higher satisfaction since specialists are chosen after isolating the medical problem and then selected by their specific skills and quality [32–34].

### Strengths and limitations

This analysis is based on a large, prospectively collected sample of PCPs. The collection of data was distributed through three different seasons; the season itself did not significantly influence the referral rate, indicating good representativeness of our data. The healthcare situation in Switzerland allows a freedom of choice of healthcare plans, allowing a direct comparison of different systems within one country. However, there are some limitations to be acknowledged: First, our study is based on a cross-sectional design and we therefore are unable to describe longitudinal data and complete patient pathways, particularly we cannot assess how many patients seek direct specialist care in non-managed health care plans. Second, our data describes the number of referrals and factors influencing the referral rate. Our study does not allow any conclusions on the optimal referral rate or any interpretation on quality of care.

## Conclusions

Patients in managed health care plans were more likely to be referred than patients without a gatekeeping insurance model. This effect remained statistically significant after correcting for potential confounders. These findings contradict the fear of a limited healthcare access for patients insured in managed care models. Referral rate and age showed a non-linear correlation with lower referral probabilities in older patients. This study underlines the central role of PCPs as coordinator of care.

## Supporting information

S1 File Data file underlying all presented findings. (XLSX)
